# Hydroxysafflor Yellow A improves diabetic nephropathy by inhibiting PI3K/AKT/mTOR pathway based on a multidimensional study

**DOI:** 10.3389/fmed.2026.1747346

**Published:** 2026-02-02

**Authors:** Jie Liu, Jie Gao, Zhibin Jiang, Wen Li, Ping Zhang, Xingde Liu, Bingqing Lyu

**Affiliations:** 1 Department of Basic Medical College, Guizhou University of Traditional Chinese Medicine, Guiyang, Guizhou, China; 2 Department of Cardiology, Guizhou Provincial People's Hospital, Guiyang, Guizhou, China; 3 Department of Cardiology, The Second Affiliated Hospital of Guizhou University of Traditional Chinese Medicine, Guiyang, Guizhou, China

**Keywords:** diabetic nephropathy, Hydroxysafflor Yellow A, *in vivo*, molecular dynamics, network pharmacology

## Abstract

**Background:**

Diabetic nephropathy (DN) remains a global health burden. This study integrates multiple approaches to investigate the therapeutic effects of Hydroxysafflor Yellow A (HSYA) in DN.

**Methods:**

SwissTargetPrediction and PharmMapper were used to predict HSYA targets. GeneCards and OMIM databases were used to identify targets associated with DN. The STRING database was used to construct the protein–protein interaction (PPI) network of key targets, and Cytoscape was applied to identify the core targets within the PPI network. GO and KEGG enrichment analyses of key targets were performed using the Metascape database. Molecular docking analyses of HSYA with core targets were performed using AutoDock Vina. The DN model was established using db/db mice fed a normal diet, with db/m mice serving as controls. Renal fibrosis was assessed by immunohistochemistry, and qPCR detected core targets and key signaling pathways.

**Results:**

We identified 236 key targets. GO and KEGG analyses were significantly enriched in the PI3K-Akt, Ras, AGE-RAGE, FoxO, mTOR, and autophagy signaling pathways. HSYA exhibited strong binding affinity with AKT1, PI3K, and mTOR. *In vivo* studies showed that HSYA modulated the expression of autophagy-related genes (PI3K, AKT, mTOR) and alleviated renal fibrosis in DN mice.

**Conclusion:**

This study provides preliminary evidence that HSYA may alleviate DN by improving renal fibrosis and modulating autophagy, thereby establishing a theoretical basis for its development as a potential therapeutic agent.

## 1 Introduction

Diabetic nephropathy (DN) is one of the most severe microvascular complications of diabetes and a leading cause of end-stage renal disease (1). The global burden of disease report has indicated that the global prevalence of diabetic nephropathy (DN) has reached 107.6 million cases as of 2021. Although age-standardized prevalence has declined by 5.1% since 1990, the disease burden has increased substantially. Age-standardized mortality rates and disability-adjusted life years due to DN have risen sharply by 37.8 and 24%, respectively, since 1990. These findings indicate that the relative incidence of DN has been partially controlled, while its global mortality and disability rates have continued to worsen, posing a serious public health challenge (2). Previous studies have demonstrated that autophagy dysregulation and fibrosis play a critical role in DN progression. Both have interacted within the molecular network of DN, where autophagy impairment has promoted extracellular matrix accumulation and interstitial remodeling by enhancing inflammatory and profibrotic signaling, ultimately leading to progressive renal dysfunction (3). The PI3K/Akt/mTOR pathway has regulated this pathological process and served as a key link between autophagy dysregulation and fibrosis (4). Clinically, overt proteinuria is a key diagnostic indicator of renal injury in patients with diabetes (5). Conventional treatment of diabetic nephropathy focuses on controlling the “three highs” (blood glucose, blood pressure, and blood lipids) and managing associated proteinuria. Recent clinical guidelines have strongly emphasized renal protection and have highlighted the importance of organ-protective agents in the management of DN (6).

Angiotensin II receptor blockers (ARBs) have held a central role in the treatment of diabetic nephropathy, as recommended by the American Diabetes Association (ADA, 2025) and Kidney Disease Improving Global Outcomes (KDIGO, 2022) guidelines. They have inhibited the renin-angiotensin system (RAS) and blocked Ang II-mediated AT1 receptor activation, which has reduced renal fibrosis and inflammation and mitigated glomerular hyperfiltration-induced damage (7). Valsartan, a widely used ARB, has demonstrated significant renal protective effects and improvement of kidney function in clinical studies (8, 9). Nonetheless, its blood pressure-lowering effect and tendency to elevate serum potassium remain major constraints on clinical application. Therefore, it is particularly important to explore potential drugs with similar or superior renal protective effects.

Hydroxysafflor yellow A (HSYA, CAS: 78281–02-4) is a quinochalcone flavonoid isolated from safflower (*Carthamus tinctorius* L.) (10). It is the principal bioactive pigment of safflower and exhibits diverse pharmacological activities (11). Previous studies have shown that it exerts therapeutic effects in neurological disorders, cardiovascular diseases, diabetes, liver fibrosis, and cancer (12). However, the regulation of autophagy and the anti-fibrotic effects by HSYA are the main focus of this study. Studies have shown that HSYA has ameliorated liver fibrosis by inhibiting HSC-mediated pro-fibrotic and pro-angiogenic processes through modulation of the miR-29a-3p/PDGFRB axis (13). Pulmonary fibrosis has been mitigated by reducing collagen deposition in the lungs of mice through modulation of the MAPK-p38 and TGF-β/Smad signaling pathways (14). The expression of TGFβ1 and P-Smad2/3 has been markedly inhibited, the NLRP3 pathway suppressed, and the accumulation of type I and type III collagen reduced, thereby ameliorating myocardial fibrosis (15). HSYA has also been reported to regulate autophagy in other diseases. It has been shown to activate neuronal autophagy via the AMPK/mTOR axis, thereby reducing inflammatory damage and facilitating neural functional recovery (16). HSYA has exerted neuroprotective effects by inducing autophagy through activation of the HIF1A/BNIP3 signaling pathway (17). By modulating the mTOR pathway, autophagy has been activated and the NLRP3 inflammasome inhibited, thereby mitigating myocardial ischemia–reperfusion injury (18). In cancer, HSYA has promoted autophagy by upregulating Beclin 1 expression and inhibiting ERK phosphorylation, resulting in HepG2 cell death (19).

To date, research on HSYA in DN has been at an early stage, with only one preliminary study reporting its protective effects through modulation of oxidative stress (20). Autophagy dysregulation and fibrosis are recognized as key pathological mechanisms underlying DN. However, whether HSYA may influence DN progression through the autophagy-fibrosis interplay remains unclear. Therefore, this is the first study to explore the potential involvement of the autophagy-fibrosis process in the nephroprotective effects of HSYA. It aims to fill an important gap in the current literature and provide a theoretical basis for further mechanistic and translational investigation.

## 2 Materials and methods

### 2.1 Identification of key targets between HSYA and DN

The chemical formula and 3D structure of HSYA were retrieved from PubChem^1^. The chemical formula was submitted to SwissTargetPrediction^2^ for target prediction, and all predicted targets were included. The 3D structure was uploaded to PharmMapper^3^ for target prediction, with the maximum number of targets set to 500 and screening restricted to the Human Protein Targets Only dataset. All targets were standardized to gene symbols using the UniProt database^4^ (*Homo sapiens*). The two datasets were merged and deduplicated to obtain the final set of predicted targets for HSYA. DN targets were retrieved from the GeneCards^5^ and OMIM^6^ databases. They were then standardized using UniProt (*Homo sapiens*). After merging and deduplication, the resulting dataset constituted the final set of predicted DN targets. The intersection of HSYA and DN targets was defined as the potential key targets mediating the effects of HSYA on DN.

### 2.2 Functional enrichment analysis

Key targets were subjected to functional enrichment analysis using Metascape^7^ (*Homo sapiens*), including Gene Ontology (GO) and KEGG pathway analyses, with a significance threshold of *p* < 0.01 (21). GO analysis included biological processes (BP), cellular components (CC), and molecular functions (MF). Key pathways and biological functions directly linked to both the compound and the disease were subsequently identified. Data visualizations were generated using publicly available online platforms (22).

### 2.3 Identification of key targets in protein–protein interaction (PPI) network

Key targets were uploaded to STRING^8^ (*Homo sapiens*) with an interaction score threshold of 0.4 to construct the PPI network. The network was subsequently imported into Cytoscape (v3.10.3) software for topological analysis (23). The cytoNCA plugin was used to calculate the betweenness and degree centrality of PPI network, and core targets were identified based on these analyses (24).

### 2.4 Molecular docking of HSYA with core targets

Molecular docking was performed using AutoDock Vina (v1.2.6) software, and the docking results were visualized with PyMOL (v3.1.5) software (25). Key interactions at the binding sites were analyzed and illustrated using PLIP database (26). The 3D structure of HSYA was retrieved from the PubChem^9^ as the ligand and imported into Chem3D (v19.0.1) software. The ligand was energy minimized using the merck molecular force field (MMFF94) (27). The AlphaFold database was used to predict the 3D structures of the core targets, which were designated as receptors (28). All ligands and receptors were further processed by adding hydrogens and performing charge balancing. Binding free energies less than −5.0 kcal/mol indicated strong receptor-ligand affinity (29).

### 2.5 Molecular dynamics of HSYA with core targets

Molecular dynamics simulations used GROMACS 2022 software. Force field parameters were obtained using the pdb2gmx tool in GROMACS and the AutoFF platform. Molecular parameters of the receptor and ligand were determined using the AMBER14SB and GAFF2 force fields, respectively. The system was solvated with TIP3P water molecules in a cubic box with a 1-nm margin (30). Ions (0.15 M NaCl) were added to the system using the gmx genion tool to ensure overall charge neutrality. Long-range electrostatic interactions were treated using the Particle Mesh Ewald method with a cutoff distance of 1 nm. All bond constraints were applied using the LINCS algorithm. Before the production simulation, the system was energy-minimized using 3,000 steps of steepest descent followed by 2,000 steps of the conjugate gradient method. The optimization steps included: (1) restraining the atoms of the receptor-ligand complex and minimizing the energy of surrounding water molecules; (2) restraining counterions and minimizing the energy of the system; (3) minimizing the energy of the entire system without any restraints. The simulation was conducted for 100 ns in the NPT (isothermal-isobaric) ensemble with a 2-fs integration step. Temperature was maintained at 310 K using the Nosé-Hoover thermostat, and pressure was kept at 1 bar using the Parrinello-Rahman barostat. System stability and conformational changes were analyzed using the GROMACS built-in tools gmx rms, gmx rmsf, gmx hbond, gmx gyrate, and gmx sasa to calculate the root-mean-square deviation (RMSD), root-mean-square fluctuation (RMSF), hydrogen bonds, radius of gyration (Rg), and solvent-accessible surface area (SASA), respectively. Gibbs free energy was evaluated using gmx sham.

### 2.6 *In vivo* analysis

#### 2.6.1 Animals

Twenty-five db/db mice (25 ± 2 g) and 5 db/m mice (23 ± 2 g), all male SPF-grade mice aged 8 weeks on a C57BL/KsJ background, were purchased from Chengdu Yaokang Biotechnology Co., Ltd. [SCXK (chuan) 2020–0034]. Mice were housed in the SPF facility at Guizhou University of Traditional Chinese Medicine under controlled conditions (temperature 20 ± 4 °C, humidity 50–60%, and 12 h light/dark cycle) and had free access to food and water. This study was approved by the Ethics Committee of Guizhou University of Traditional Chinese Medicine (No. 20241109002) and conducted in strict accordance with the ARRIVE guidelines as well as the *Guide for the Care and Use of Laboratory Animals* (31).

#### 2.6.2 Drugs, chemicals, and equipment

HSYA (T3674, TargetMol, Boston, United States). Valsartan (Novartis, Switzerland). Antibodies and reagents used in this study included α-SMA antibody (19,245, CST, Boston, United States); FN antibody (E-AB-2077, Elabscience, Wuhan, China); E-cadherin antibody (20874-1-AP, Proteintech, Wuhan, China); HRP-conjugated goat anti-rabbit secondary antibody (BA1054, Boster, Wuhan, China); HRP-conjugated goat anti-mouse secondary antibody (BA1051, Boster, Wuhan, China); TRIzol (15596–026, Ambion, Austin, United States); HiScript^®^ II Q RT SuperMix for qPCR (R223-01, Vazyme, Nanjing, China); SYBR Green Master Mix (Q111-02, Vazyme, Nanjing, China); and Taq Plus DNA Polymerase (ET105-02, Tiangen, Beijing, China). Equipment included desktop high-speed refrigerated centrifuge (H1-16KR, Hunan Kecheng Instruments Co., Ltd., China); transmission electron microscope (HT-7700, Hitachi, Tokyo, Japan); chemiluminescence imaging system (PR-96, Hangzhou Shenhua Technology Co., Ltd., China); multimode microplate reader (Varioskan LUX, Thermo, Waltham, United States); real-time PCR system (PR-96, Hangzhou MEO Instruments Co., Ltd., China); vortex mixer (Scientific Industries, Thermo, Germany); and laser confocal scanning microscope (Leica TCS-SP2, Wetzlar, Germany).

#### 2.6.3 Experimental design

All mice (8 weeks old) underwent 1 week of adaptation and began the intervention at 9 weeks of age. The intervention lasted for 8 weeks, and the last intervention was performed on the final day at 16 weeks of age. The db/m mice served as the control group (n = 5). The db/db mice were randomly assigned to the following 5 groups (n = 5 per group) based on body weight: model (db/db), low-dose (db/db + HSYA 2.5 mg/kg), medium-dose (db/db + HSYA 5 mg/kg), high-dose (db/db + HSYA 10 mg/kg), and positive control (db/db + valsartan 10.4 mg/kg). Random assignment was performed using a computer-generated randomization procedure. Each treatment group and the positive control group received oral gavage once daily for 8 weeks, while the control and model groups were given an equal volume of physiological saline. Based on the db/db genotype, mice exhibited hyperglycemia and early renal dysfunction from 8 weeks of age and developed a stable DN model by 16 weeks (32). All mice completed the study and no animals were excluded. All histological and quantitative data were labeled using sequential numbers instead of group identifiers. Tissue and data acquisition were performed by investigators (Z. J. and B. L.), who were not involved in data validation or statistical analysis. All data validation and formal analyses were independently conducted by other investigators (J. L., J. G., and W. L.) under blinded conditions to minimize potential bias. On the first day at 17 weeks of age, all mice were individually housed in metabolic cages and fasted with free access to water. Twenty-four hours urine samples were collected, centrifuged at 3,000 × *g* for 10 min at 4 °C, and the supernatant was stored at −20 °C until analysis. Urinary albumin (ALB) concentrations were measured using an ELISA kit in accordance with manufacturer instructions. Finally, all mice were anesthetized with an intraperitoneal injection of 1% pentobarbital sodium (50 mg/kg). After confirming the loss of pedal and other reflexes, cervical dislocation was performed by trained personnel to achieve euthanasia, and animal death was verified by the cessation of respiration and heartbeat (33). Terminal blood was then collected by ocular enucleation and kidney tissues were subsequently harvested for further analysis. Blood was allowed to clot for 2 h, then centrifuged at 3,000 × g for 10 min at 4 °C. Serum was collected from the supernatant and stored at −80 °C for later use. Both kidneys were excised, and surrounding fat and fascia were removed. The tissues were rinsed with saline, blotted dry, and weighed. Portions were cut in the sagittal plane and fixed in 4% paraformaldehyde for histopathology. The remaining tissue was stored at −80 °C.

#### 2.6.4 Fasting blood glucose (FBG)

Mice were fasted for 6–8 h before testing and fasting blood glucose was measured using a glucometer after tail vein puncture with a disposable lancet. FBG was recorded before the first administration and after the final dose.

#### 2.6.5 Renal histology and hematoxylin and eosin (H&E) staining

Kidney tissues from each mouse group were embedded in paraffin and pretreated (sectioned, deparaffinized, and washed with PBS), then stained with H&E to observe pathological changes under a light microscope.

#### 2.6.6 Immunohistochemical (IHC) analysis

Kidney tissues were pretreated (sectioned, deparaffinized, and washed with PBS) and incubated sequentially with 3% H₂O₂ to block endogenous peroxidase (15 min), normal goat serum for blocking (30 min), and primary antibodies at 4 °C overnight (*α*-SMA, 1:400; FN, 1:200; E-cadherin, 1:200). Sections were then incubated with HRP-conjugated secondary antibodies (37 °C, 30 min), developed with DAB until the desired staining intensity was reached, and counterstained with hematoxylin (2 min). After dehydration in ethanol and clearing in xylene, slides were mounted with neutral resin and imaged under a microscope. IHC images were quantitatively analyzed using Image-Pro Plus 6.0 software (Media Cybernetics, United States). Four random fields per section were captured and analyzed at 400 × magnification. The area, integrated optical density (IOD), and mean density of positive staining regions were calculated, with mean density representing the relative protein expression level.

#### 2.6.7 RNA extraction and quantitative PCR

Total RNA was extracted from each group using the Trizol method. Next, RNA was reverse transcribed into cDNA following the standard protocol of the HiScript^®^ II Q RT SuperMix for qPCR kit. The real-time quantitative PCR was performed following the instructions of the SYBR Green Master Mix. The reaction conditions were as follows: (1) Initial denaturation occurred at 95 °C for 10 min. (2) The amplification consisted of 40 cycles, each with denaturation at 95 °C for 15 s, annealing at 60 °C for 30 s, and extension at 60 °C for 30 s. (3) A melting curve was generated from 65 °C to 95 °C with fluorescence signals collected at 0.5 °C intervals. Data were analyzed using the 2^–ΔΔCt^ method. Primer sequences used in this study were listed in Table 1.

**Table 1:** Gene primer information.

Gene	Primer	Sequence (5′-3′)	PCR products
Mus β-actin	Forward	CCAGCCTTCCTTCTTGGGTAT	103 bp
	Reverse	GTTGGCATAGAGGTCTTTACGG	
Mus PI3K	Forward	AATGCACGGCGATTACACTC	199 bp
	Reverse	GGACACTGGGTAGAGCAACT	
Mus AKT	Forward	CTGCCCTTCTACAACCAGGA	214 bp
	Reverse	CATACACATCCTGCCACACG	
Mus mTOR	Forward	AGAACTTGGAGAACCAGCCC	199 bp
	Reverse	CAGCTCCACTTGGGTTGGAA	

### 2.7 Statistical analysis

Statistical analyses were conducted using GraphPad Prism 8.0. All experimental data were expressed as mean ± standard deviation (SD). Differences among groups were assessed by one-way ANOVA followed by Dunnett’s T3 *post hoc* test for multiple comparisons between groups. *p* < 0.05 was considered statistically significant.

## 3 Results

### 3.1 Identification of key targets

**Figure 1:**
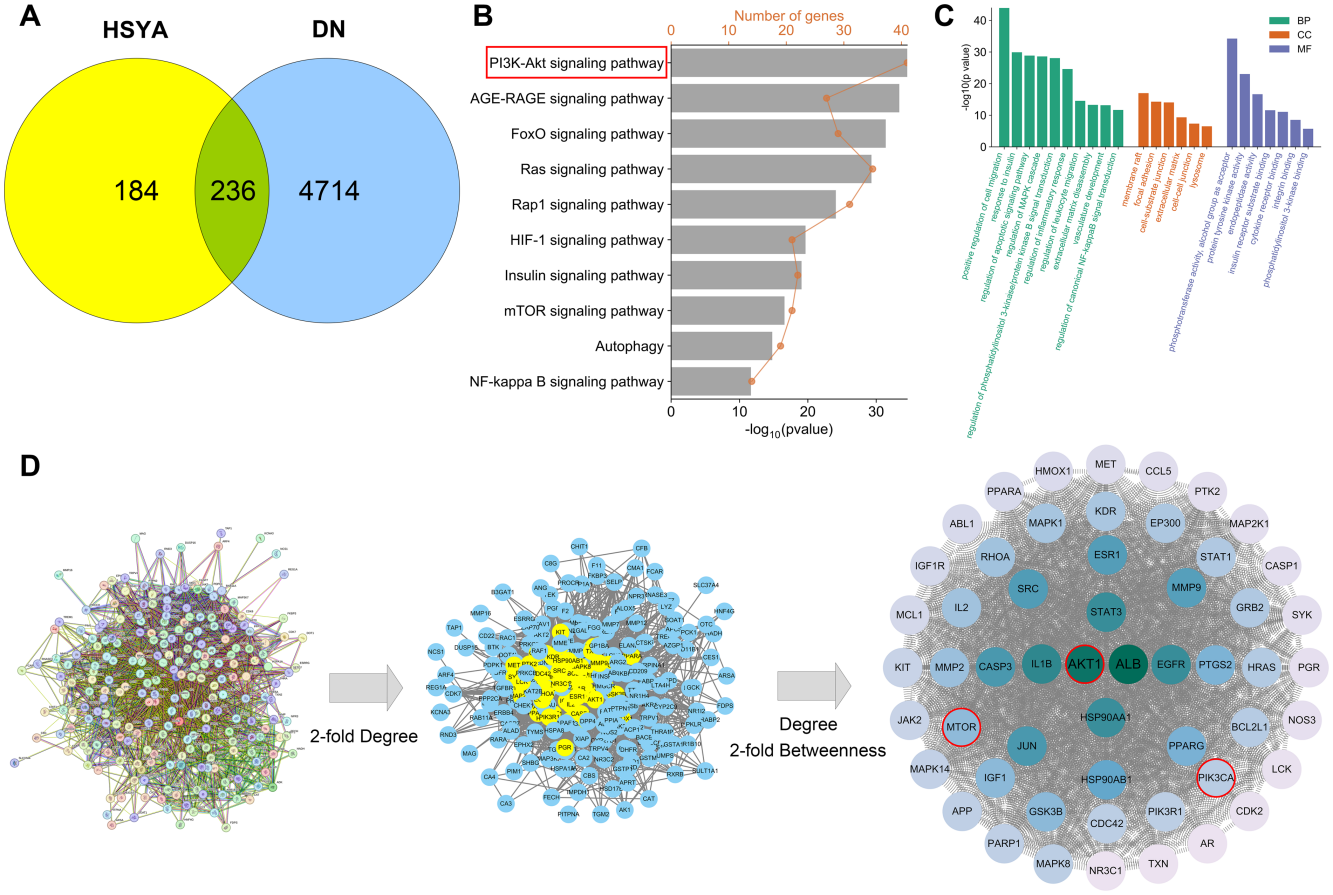
Results of hub gene screening and functional enrichment analysis. **(A)** Identification of compound and disease targets. **(B)** PPI network and hub gene screening of key targets. **(C)** KEGG pathway enrichment analysis of key targets. **(D)** GO functional enrichment analysis of key targets. PPI, protein–protein interaction; GO, gene ontology; KEGG, Kyoto Encyclopedia of Genes and Genomes; BP, biological processes; CC, cellular components; MF, molecular functions; HSYA, Hydroxysafflor Yellow A; DN, diabetic nephropathy.

Through searches and identification in public databases, we obtained 420 potential targets of HSYA and 4,950 targets associated with DN. This study intersected the HSYA and DN target sets, identifying 236 shared targets, which were defined as key targets of HSYA in the treatment of DN (Figure 1A). These key targets were used in subsequent analyses.

### 3.2 Results of functional enrichment analysis

The GO and KEGG enrichment analyses were performed on the key targets. The results revealed significant enrichment of 10 signaling pathways, including PI3K-Akt, Ras, AGE-RAGE, FoxO, HIF-1, insulin, mTOR, NF-κB, and autophagy pathways (Figure 1B). This suggested that HSYA most likely acted by modulating core mechanisms of fibrosis, inflammatory response, glucose metabolism, vascular function, and apoptosis. BP analysis showed that HSYA regulated cell survival and anti-apoptotic signaling, suppressed chronic inflammatory responses, and modulated cell migration and extracellular matrix accumulation, thereby improving cellular injury and tissue fibrosis in DN. CC analysis suggested that HSYA counteracted DN-induced cellular damage and tissue fibrosis by stabilizing membrane rafts and lysosomes, maintained the integrity of the cell substrate and intercellular junctions, and modulated extracellular matrix remodeling. MF analysis focused on key enzymatic and binding functions, showed that HSYA modulated phosphotransferase and protein tyrosine kinase activity to influence cell signaling, and regulated cytokine receptor and integrin binding to affect cell communication and adhesion, ultimately impacted DN pathogenesis (Figure 1C).

### 3.3 Hub genes from PPI network

Key targets were imported into the STRING database to construct a PPI network comprising 236 nodes and 3,711 edges, with highly significant enrichment (*p* < 1.0 × 10^−16^). Subsequently, Cytoscape was used to extract a subnetwork based on a 2-fold median degree centrality threshold. Finally, the subnetwork was further screened using degree centrality and a 2-fold median betweenness centrality threshold. This process yielded 54 core targets, including AKT1, PIK3CA and mTOR (Figure 1D).

### 3.4 Molecular docking

**Figure 2:**
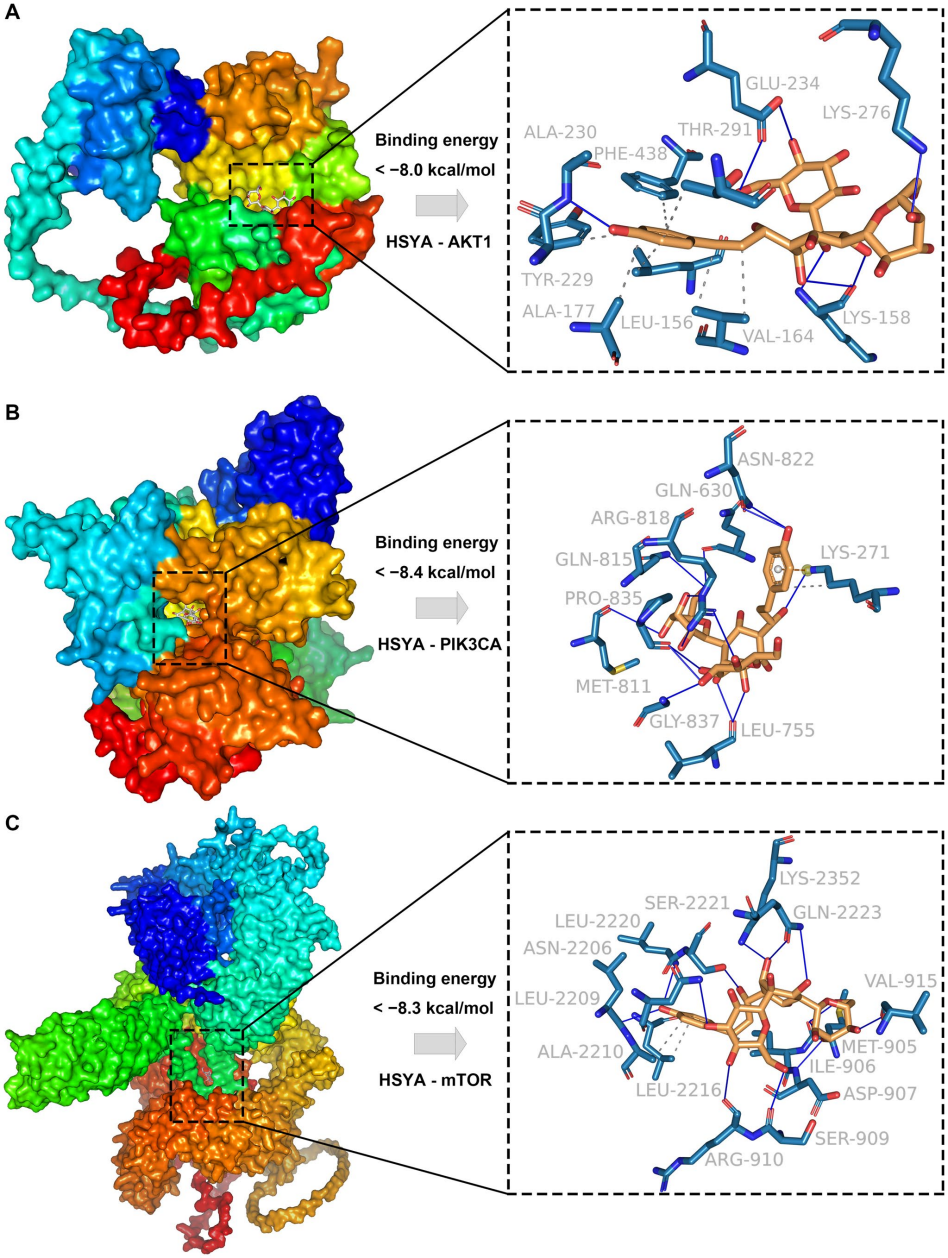
Molecular docking results of HSYA and hub genes. **(A)** Binding conformation of HSYA and AKT1 protein. **(B)** Binding conformation of HSYA and PIK3CA protein. **(C)** Binding conformation of HSYA and mTOR protein. Blue solid lines represent hydrogen bonds, and gray dashed lines represent hydrophobic interactions.

The results indicated that HSYA showed strong binding affinity to AKT1, PIK3CA and mTOR, with binding energies less than −8.0 kcal/mol. For AKT1, the receptor-ligand complex exhibited high stability, primarily driven through 7 hydrophobic interactions formed by 6 residues (LEU-156, VAL-164, ALA-177, TYR-229, THR-291, PHE-438) and 8 hydrogen bonds formed by 4 residues (LYS-158, ALA-230, GLU-234, LYS-276) (Figure 2A). For PIK3CA, complex stability was maintained through 13 hydrophobic interactions formed by 8 residues (GLN-630, LEU-755, MET-811, GLN-815, ARG-818, ASN-822, PRO-835, GLY-837) and three additional interactions (*π*-cation interaction, hydrogen bond, and hydrophobic interaction) involving LYS-271 (Figure 2B). For mTOR, complex stability was primarily maintained through 3 hydrophobic interactions formed by 3 residues (ILE-906, ALA-2210, LEU-2216) and 13 hydrogen bonds involving 11 residues (MET-905, ASP-907, SER-909, ARG-910, VAL-915, ASN-2206, LEU-2209, LEU-2220, SER-2221, GLN-2223, LYS-2352) (Figure 2C).

### 3.5 Molecular dynamics

**Figure 3:**
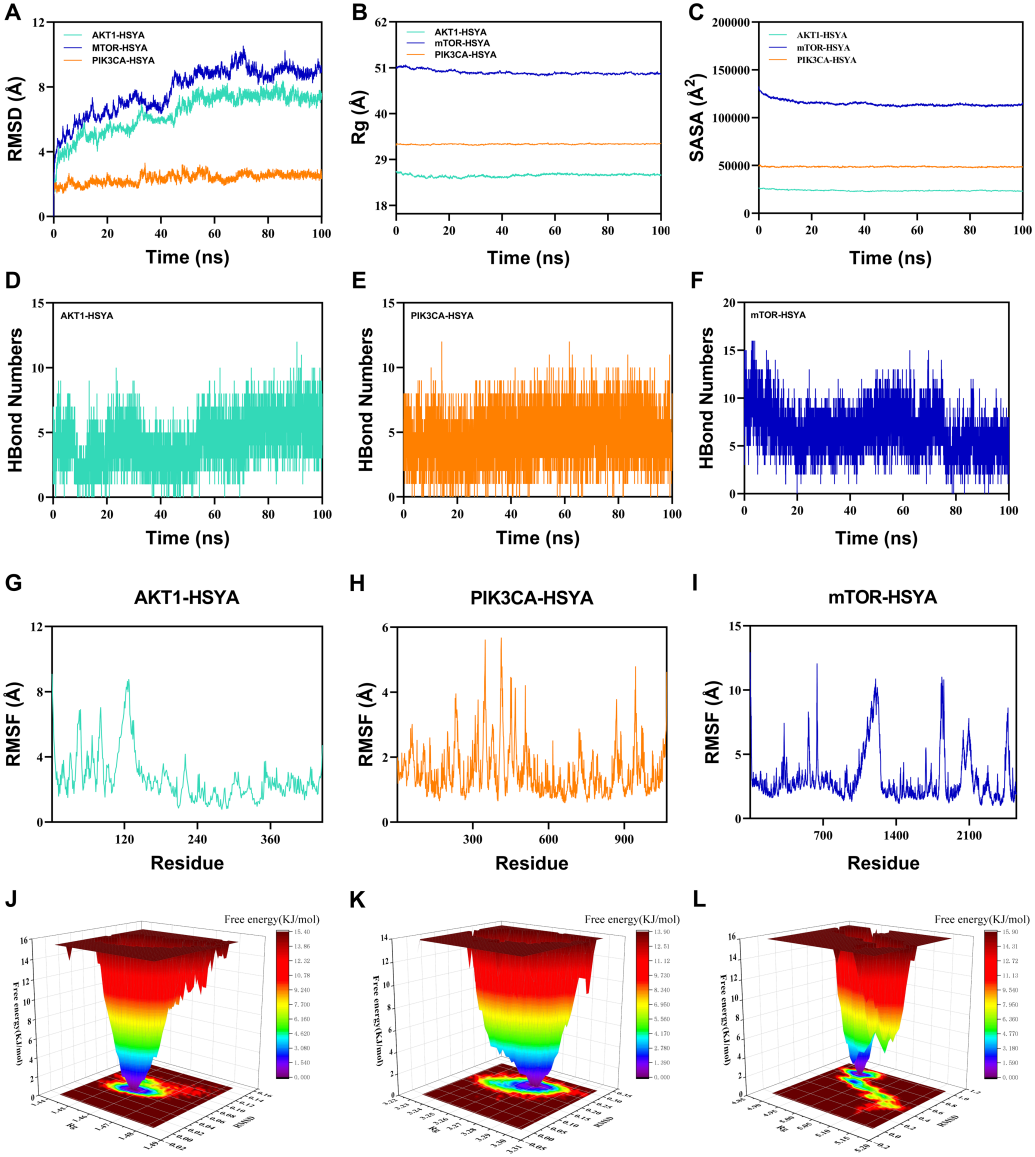
Molecular dynamics results of the protein-compound complexes. **(A)** RMSD analysis of protein-HSYA complexes over a 100 ns simulation. **(B)** Rg analysis of protein-HSYA complexes over a 100 ns simulation. **(C)** SASA of protein-HSYA complexes during molecular dynamics. **(D)** Time-dependent hydrogen bond fluctuations in the AKT1-HSYA complex. **(E)** Time-dependent hydrogen bond fluctuations in the PIK3CA-HSYA complex. **(F)** Time-dependent hydrogen bond fluctuations in the mTOR-HSYA complex. **(G)** RMSF analysis of the AKT1-HSYA complex. **(H)** RMSF analysis of the PIK3CA-HSYA complex. **(I)** RMSF analysis of the mTOR-HSYA complex. **(J)** FEL of the AKT1-HSYA complex. **(K)** FEL of the PIK3CA-HSYA complex. **(L)** FEL of the mTOR-HSYA complex. RMSD, Root Mean Square Deviation; Rg, Radius of Gyration; SASA, Solvent Accessible Surface Area; RMSF, Root Mean Square Fluctuation; FEL, Free Energy Landscape.

The RMSD assessed the conformational stability of the ligand and receptor (Figure 3A). The AKT1-HSYA complex equilibrated after 50 ns, fluctuating around 7.5 Å, and the PIK3CA-HSYA complex equilibrated after 60 ns, fluctuating around 2.4 Å. The mTOR-HSYA complex showed a rising trend during the simulation, fluctuating around 9 Å. These results indicated that HSYA maintained high stability with AKT1 and PIK3CA, while its interaction with mTOR showed comparatively more variation. Rg was used to assess changes in the overall structure (Figure 3B). The PIK3CA-HSYA complex exhibited stable fluctuations during the simulation. The AKT1-HSYA and mTOR-HSYA complexes displayed minor fluctuations and underwent slight conformational changes throughout the process. SASA analysis was performed to evaluate conformational changes and solvent exposure of the complex system (Figure 3C). The binding of PIK3CA to HSYA showed no significant fluctuation. The AKT1-HSYA complex exhibited slight fluctuations, indicating that ligand binding altered the binding microenvironment and stabilized after 40 ns. The mTOR-HSYA system initially contracted but later reached a relative equilibrium. Hydrogen bond interaction analysis showed that the PIK3CA-HSYA complex maintained the most stable and numerous hydrogen bond network (averaging 7–8 bonds), followed by the AKT1-HSYA complex (averaging 5–6 bonds) (Figures 3D,E). The binding of mTOR and HSYA exhibited fluctuations, with an average of 4 to 8 hydrogen bonds (Figure 3F). The PIK3CA protein exhibited the lowest RMSF values (ranging from 0.5 to 2.0 Å), indicating the highest structural rigidity (Figure 3H). The overall flexibility of the AKT1 protein was moderate (ranging from 1.0 to 3.0 Å), with a major local high-flexibility region (Figure 3G). The mTOR protein exhibited the highest flexibility, with several large flexible domains (peaking at 12.0 Å), consistent with its large kinase structure (Figure 3I). Both the AKT1-HSYA and PIK3CA-HSYA systems exhibited high thermodynamic stability, with their free energy landscapes dominated by a narrow and deep free energy well (Figures 3J,K). This suggested that both complexes were rigid and stable. In contrast, the mTOR-HSYA system displayed multiple distinct free energy minima, indicating that the complex conformation was more flexible (Figure 3L).

### 3.6 Effects of HSYA on FBG and body weight

**Figure 4:**
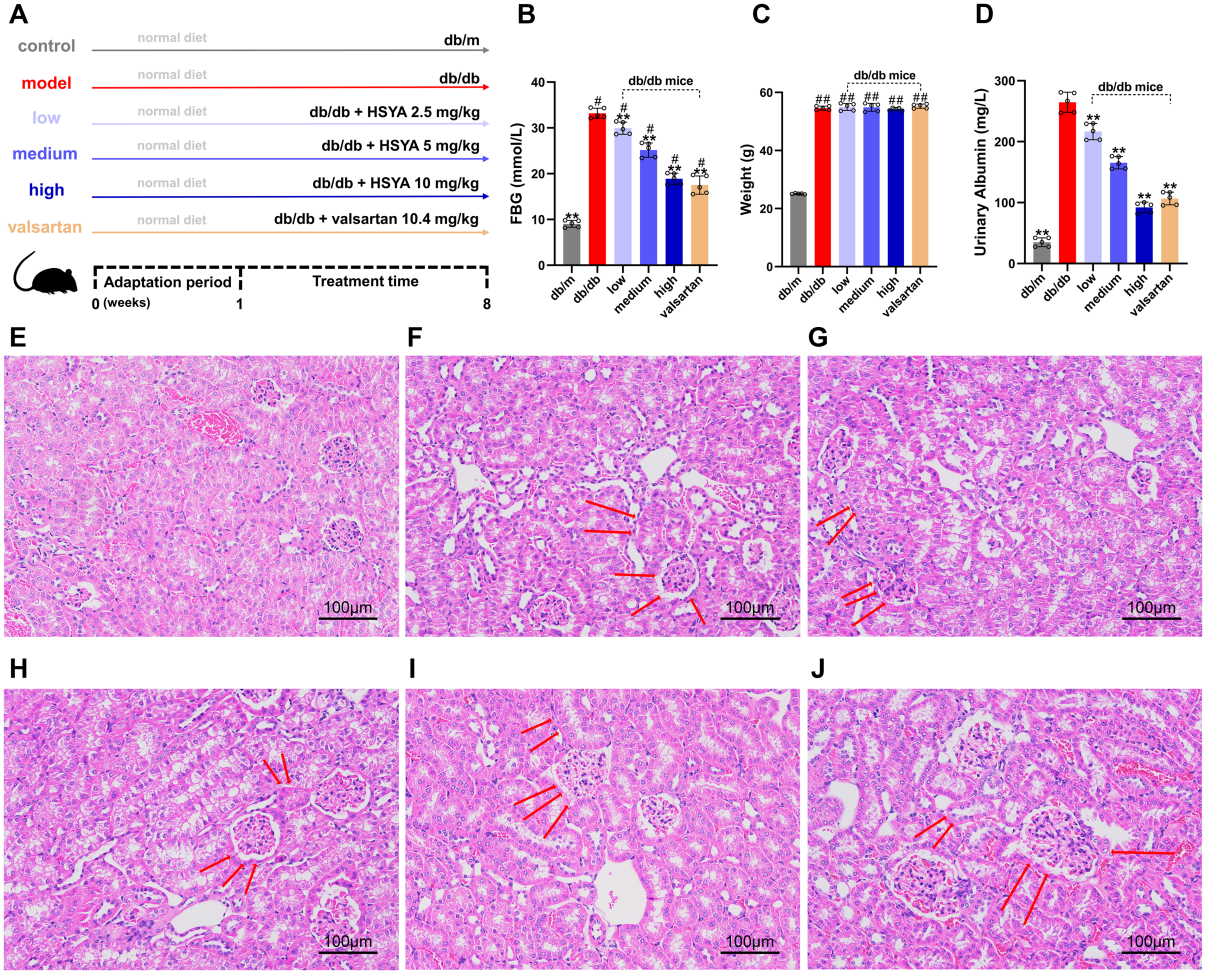
Effects of HSYA on biochemical indicators and renal histopathology (H&E staining) in DN mice (Scale bar: 100 μm). **(A)** Experimental design and treatment scheme for all groups. **(B)** FBG levels in different groups of mice. **(C)** Body weight changes in different groups of mice. **(D)** 24-h urinary albumin levels in different groups of mice. **(E)** db/m (control) group. **(F)** db/db (model) group. **(G)** db/db + HSYA 2.5 mg/kg (low) group. **(H)** db/db + HSYA 5 mg/kg (medium) group. **(I)** db/db + HSYA 10 mg/kg (high) group. **(J)** db/db + Valsartan 10.4 mg/kg (valsartan) group. Data represent means ± SD (*n* = 5 in each group). ^#^*p* < 0.05 vs. db/m group; *p < 0.05, ** *p* < 0.01 vs. db/db group. FBG, fasting blood glucose.

After a one-week adaptation period, the treatment and positive control groups were administered HSYA and valsartan, respectively (Figure 4A). After 8 weeks of feeding and treatment, the model and treatment groups exhibited significantly higher blood glucose levels than the control group (*p* < 0.01), whereas glucose levels in the treatment groups were significantly reduced compared with the model group (*p* < 0.01) (Figure 4B). Body weight was significantly increased in the model and treatment groups relative to the control group (*p* < 0.01), with no significant differences between the HSYA or valsartan groups and the model group (Figure 4C).

### 3.7 Effect of HSYA on 24-h urinary albumin levels

Compared to the control group, 24-h urinary albumin was significantly elevated in the model group (*p* < 0.01). All HSYA-treated groups showed a significant reduction in 24-h urinary albumin compared to the model group (*p* < 0.01) (Figure 4D).

### 3.8 HSYA ameliorates renal histopathological changes

The H&E staining revealed significant differences in renal pathology among the groups. The glomerular morphology in the control group was normal, with well-ordered renal tubules and no abnormalities in the interstitium (Figure 4E). The model group exhibited severe renal damage, including diffuse mesangial proliferation, glomerular swelling, tubular edema, lumen dilation, and widespread interstitial inflammatory cell infiltration (Figure 4F). Compared to the model group, the intervention groups showed the following improvements: (1) the low-dose group showed mild improvements in renal pathological damage, with slight reductions in glomerular hypertrophy and mesangial proliferation. Tubular epithelial cell swelling and vacuolar degeneration were also slightly alleviated (Figure 4G). (2) The renal pathological damage in the medium-dose group was further improved, including a significant reduction in mesangial proliferation, a more regular glomerular morphology, alleviated tubule edema, and a decrease in interstitial inflammatory cell infiltration (Figure 4H). (3) The high-dose group exhibited more significant improvements, including near-normalization of glomerular morphology, organized arrangement of renal tubules, alleviation of tubular dilation, and minimal inflammatory cell infiltration in the interstitium (Figure 4I). (4) The trend of improvement in the valsartan group was similar to that in the high-dose group, including reduced mesangial proliferation, significant alleviation of glomerular swelling, relatively intact tubular structure, and marked attenuation of interstitial inflammation (Figure 4J). The renal histopathological results indicated that HSYA intervention alleviated renal damage in a dose-dependent manner.

### 3.9 HSYA modulates fibrosis-related protein expression

**Figure 5:**
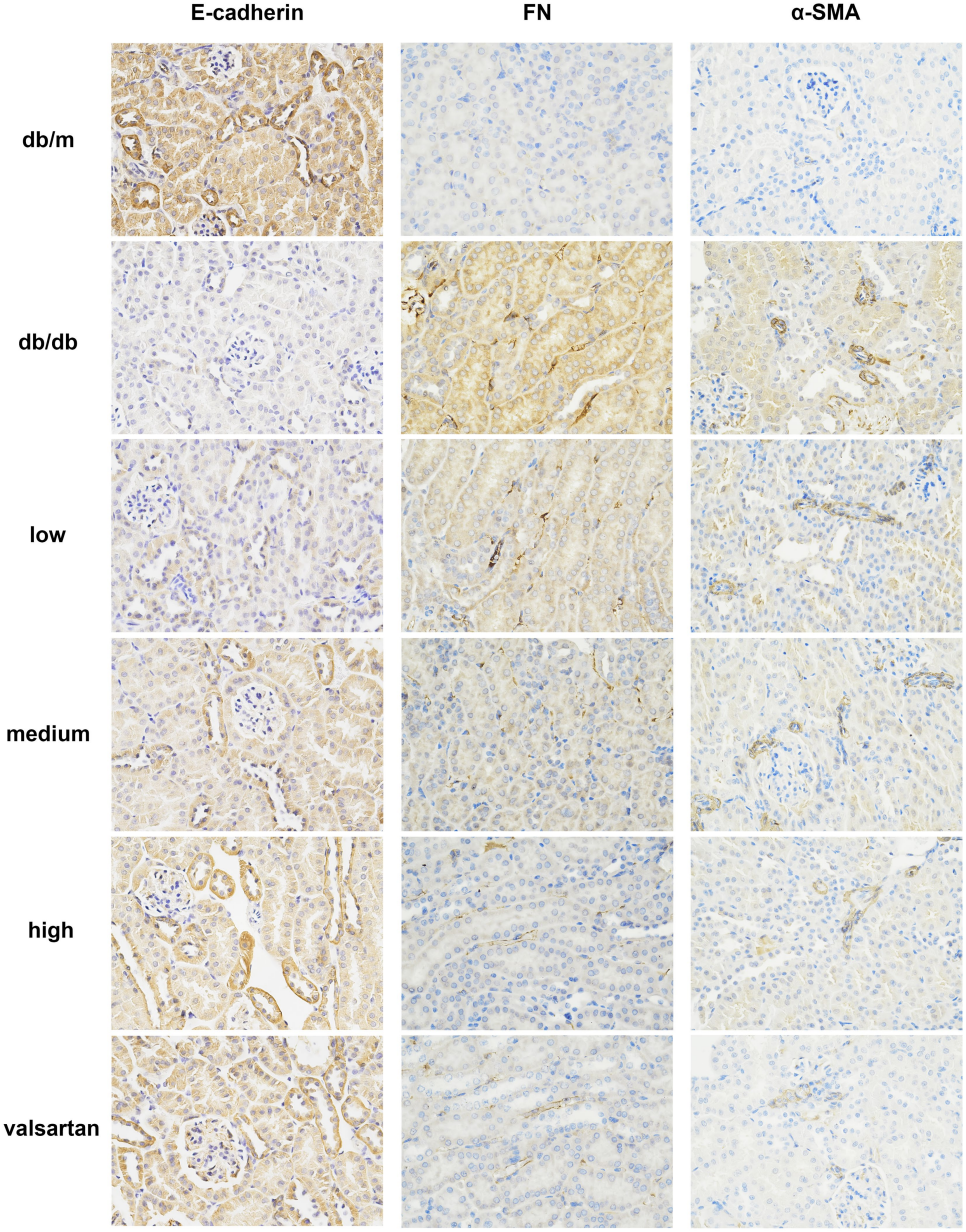
Immunohistochemical staining results of E-cadherin, FN, and α-SMA in the renal tissue from various DN mouse groups (×400). FN, fibronectin; α-SMA, α-smooth muscle actin.

**Figure 6:**
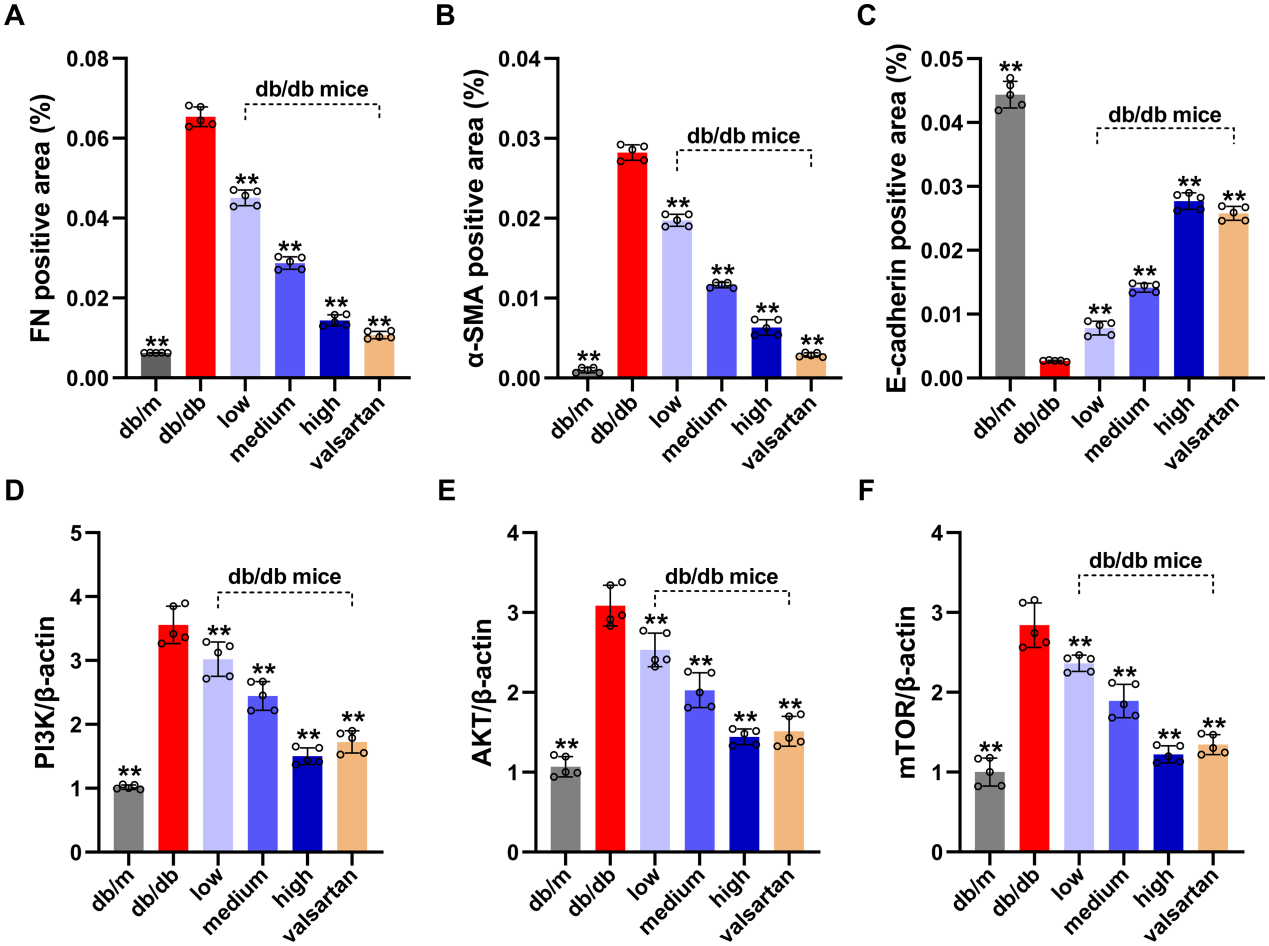
Results of EMT protein levels (quantified by IHC grayscale analysis) and PI3K/AKT/mTOR pathway gene expression (by qPCR) in the renal tissue from various DN mouse groups. **(A)** FN positive area (%). **(B)** α-SMA positive area (%). **(C)** E-cadherin positive area (%). **(D)** Relative expression of *PIK3CA* gene. **(E)** Relative expression of *AKT* gene. **(F)** Relative expression of *mTOR* gene. Data represent means ± SD (*n* = 5 in each group). *p < 0.05, **p < 0.01 vs. db/db group. IHC, immunohistochemical; FN, fibronectin; α-SMA, α-smooth muscle actin; EMT, epithelial-mesenchymal transition.

We analyzed all groups by immunohistochemistry (Figure 5). The expression of fibrosis markers (α-SMA and FN) significantly differed between the groups. In the model group, α-SMA and FN expressions were significantly upregulated (*p* < 0.01). α-SMA was widely deposited in the glomerular mesangium and renal interstitium, while FN showed strong expression in the renal interstitium. Compared to the model group, the HSYA intervention groups demonstrated a dose-dependent improvement in renal pathology (*p* < 0.01) (Figures 6A,B): (1) in the low-dose group, α-SMA and FN expressions were slightly reduced. (2) Both the medium-dose and high-dose groups exhibited significant downregulation, with the high-dose group showed the most pronounced difference. The performance of the valsartan group was similar to that of the high-dose group. The epithelial-mesenchymal transition (EMT) marker E-cadherin exhibited an opposite expression trend. Compared to the model group, the other groups exhibited significant differences (*p* < 0.01) (Figure 6C): (1) the high and medium doses of HSYA effectively maintained E-cadherin expression. (2) The low-dose group maintained partial expression. The high-dose and valsartan groups showed a similar trend in E-cadherin expression. The results suggested that HSYA dose-dependently regulated the expression of fibrosis-related proteins, thereby inhibiting the progression of fibrosis in DN.

### 3.10 HSYA modulates the PI3K/Akt/mTOR pathway

We analyzed genes highly associated with autophagy regulation by qPCR (Figures 6D–F). Compared to the model group, the mRNA levels of PI3K, Akt, and mTOR in kidney tissue from the control group were significantly reduced (*p* < 0.01). This indicated that the expression levels of these mRNAs were lower in normal kidney tissue and were markedly elevated in the kidney tissue of DN. All intervention groups showed significant inhibitory effects compared to the model group: (1) the low, medium, and high dose groups were associated with significant inhibitory effects (*p* < 0.01). (2) The HSYA treatment groups exhibited a dose-dependent inhibitory effect. (3) The high-dose group demonstrated the most significant inhibition. (4) The valsartan group also significantly inhibited the mRNA expressions of PI3K, Akt, and mTOR (*p* < 0.01), with effects comparable to the high-dose group.

## 4 Discussion

This study employed multiple approaches to elucidate the mechanisms by which HSYA ameliorated DN. The results indicated that HSYA interacted with PIK3CA, AKT1, and mTOR, thereby influencing the PI3K/AKT, fibrosis, and autophagy signaling pathways. Experimental validation demonstrated that HSYA alleviated DN-induced renal injury by improving renal fibrosis and modulating the expression of autophagy-related genes (PI3K, AKT, and mTOR).

The KEGG analysis revealed that key targets of HSYA in DN were closely associated with pathological processes, including inflammation, fibrosis, autophagy, and apoptosis. GO analysis showed that the core genes primarily participate in phosphotransferase, protein tyrosine kinase, and endopeptidase activities. Specific binding involved phosphatidylinositol 3-kinase binding, insulin receptor substrate binding, and integrin binding. In CC, they localized mainly to focal adhesions, membrane rafts, and cell-matrix junctions, which mediate interactions between cells and the microenvironment. BP terms were enriched in the regulation of PI3K/Akt signaling, positive regulation of cell migration, and regulation of apoptotic signaling pathways. This multidimensional association indicates that the gene set plays a central role in cell proliferation, survival, and metastasis. Its functional localization, combined with kinase activity, supports a mechanism whereby interactions between cells and the microenvironment activate core signaling pathways. The PI3K/Akt pathway was the most enriched and acted as a key upstream regulator of autophagy, fibrosis, and inflammation. Its downstream mTOR pathway, along with the mTOR-suppressed autophagy pathway, was also significantly enriched. Together, these pathways have formed the PI3K/Akt/mTOR signaling, which has played a central role in regulating autophagy in DN (34). Additionally, HIF-1α has regulated oxidative stress through the PI3K/AKT pathway, thereby modulating the release of profibrotic factors (35). This pathway has also functioned as a component of the DsbA-L/AKT/PGC-1α signaling axis and has played a critical role in renal aging and fibrosis (36). This conclusion has been supported by previous *in vitro* and *in vivo* studies, which have shown that downregulating PI3K, Akt, and mTOR enhances autophagy and consequently ameliorates DN (37, 38). Ras and Rap1 have been identified as classical upstream activators of PI3K/Akt, directly contributing to its aberrant activation and driving cellular proliferation and injury (39). The AGE-RAGE signaling pathway also activates PI3K/Akt through the binding of AGE ligands to RAGE receptors, leading to excessive ROS production and induction of inflammatory responses (40). Overactivation of PI3K/Akt has increased mTOR signaling, strongly suppressed autophagy (34), inactivated FoxO, relieved suppression of pro-apoptotic genes, and disrupted insulin signaling, thereby aggravating insulin resistance (41, 42). Furthermore, it has activated HIF1α signaling, enhancing HIF1α protein translation and stability, which induces hypoxic stress in renal tubulointerstitial tissue and contributes directly to renal fibrosis (43). It has synergized with NF-κB signaling, markedly upregulating inflammatory factors while accelerating renal inflammation and fibrosis (44).

Molecular docking showed that HSYA formed a highly stable complex with AKT1. This stability was primarily driven by strong hydrophobic interactions and hydrogen bonds. Previous studies have indicated that these binding patterns resemble those of potent competitive inhibitors. The inhibitor has directly competed with ATP binding by forming key hydrophobic contacts with conserved hinge residues within the ATP-binding pocket (LEU-156 and VAL-164) and establishing a strong hydrogen-bond network with the core anchoring residue LYS-158 (45). Additionally, HSYA has interacted with residues (PHE-438 and THR-291), further suggesting that it may have affected the stability of the allosteric regulatory loop or C-helix of AKT1 (46). For PIK3CA, HSYA formed a stable complex with the kinase through hydrogen bonds, hydrophobic interactions, and *π*-cation contacts. These interactions have strengthened electrostatic anchoring and stabilized the kinase–ligand interface while influencing the activation loop and potentially disrupting ATP binding, thus promoting the kinase to remain in an inactive conformation (47–49). For mTOR, the complex is primarily stabilized by extensive hydrophobic interactions and hydrogen bonds. By directly occupying the ATP-binding site and forming multiple hydrogen bonds, this mode aligns with the design strategy of previously reported ATP-competitive mTOR inhibitors (50). Molecular dynamics results showed that HSYA exhibited strong binding affinity with the receptor protein. Specifically, the PIK3CA-HSYA and AKT1-HSYA complexes exhibited high stability during the simulation, whereas the mTOR-HSYA complex showed moderate structural fluctuations after binding. We speculate that the large size of the mTOR protein and its extensive flexible regions are the primary factors contributing to the observed fluctuations after binding. Based on its favorable binding patterns and potential inhibitory effects on AKT1, PIK3CA, and mTOR, HSYA demonstrates strong potential as a multi-target inhibitor of the PI3K/AKT/mTOR signaling pathway.

HSYA (C₂₇H₃₂O₁₆) was first reported in 1981, and its chemical structure was identified in 2013 (51). It is water soluble but unstable under light, heat, and alkaline conditions. It has been used as a quality control standard because of its high content and strong bioactivity in safflower (52). HSYA has been primarily absorbed in the small intestine through passive diffusion and has reached its peak plasma concentration within approximately 10 min after oral administration (53–55). However, it has scarcely penetrated the intact blood–brain barrier and has been excreted mainly in urine (48%) and slightly in feces (2.9%) (56, 57). Notably, studies have shown that HSYA has a low plasma protein binding rate and does not engage in competitive binding with other drugs, indicating a high clinical safety profile (58). HSYA has exhibited high polarity and poor penetration of the phospholipid bilayer, resulting in low oral bioavailability (57). Strategies to enhance the bioavailability of HSYA have achieved significant progress: compared with HSYA solution, HSYA microemulsions have effectively improved molecular permeability (59), solid lipid nanoparticles have increased oral bioavailability by approximately 3.97-fold (60); co-administration with 0.02 mg/mL *Ligusticum* chuanxiong volatile oil has raised bioavailability 6.48-fold (57); HSYA-phospholipid complexes have increased bioavailability to 37-fold (61). Recent studies have elucidated the biosynthetic pathway of HSYA and its key enzymes, providing a clear technical foundation for the sustainable, large-scale, and low-cost bioproduction of this valuable medicinal natural product and demonstrating significant industrial potential (62).

Although HSYA demonstrates potential therapeutic effects in multiple diseases, clinical evidence for its use in kidney diseases remains lacking. However, findings from phase I and phase II clinical trials of HSYA in acute ischemic stroke have provided important translational evidence. Results from two Phase I clinical trials have indicated that HSYA has demonstrated favorable pharmacokinetics (median *T*_max_ of 1.1 h and t_1/2_ of 4.0–4.7 h), good tolerance, and few adverse events (63, 64). In a phase II trial, the proportions of patients achieving a Modified Rankin Scale score ≤ 1 have been significantly higher in the medium- and high-dose HSYA groups compared with the control group. The rates of favorable outcomes defined by National Institute of Health Stroke Scale score ≤ 1, Barthel Index score ≥ 95, and blood stasis syndrome score ≥ 30% have also been significantly higher in these groups (65). A meta-analysis of 31 RCTs on DN has shown that safflor yellow pigment formulation containing HSYA as the main component improves proteinuria, kidney function, blood glucose, blood lipids, inflammation, and oxidative stress (66). Although these studies offer valuable insights into the clinical efficacy of HSYA, caution is warranted in interpreting the results due to the use of a mixed formulation rather than a pure monomer. To support the clinical translation and precision medicine application of HSYA as an independent therapeutic agent, further research is needed to investigate its pharmacological mechanisms and clinical trial.

In recent years, growing evidence has demonstrated that traditional medicine plays a significant role in the management of kidney diseases. Salidroside has exerted antioxidant and anti-aging effects via multiple pathways and targets, contributing to the prevention of DN and its complications (67). Qufeng Tongluo decoction has demonstrated a unique effect in ameliorating podocyte injury by regulating P62 expression and enhancing podocyte autophagy, thereby promoting recovery from damage (68). Danggui Buxue Tang has exhibited significant therapeutic efficacy in renal anemia by modulating the PI3K-Akt and FoxO signaling pathways (69). *Rheum officinale* and its active constituents have been shown to exert significant therapeutic effects by suppressing renal fibrosis and inflammatory responses (70). In the management of acute kidney injury and chronic kidney disease, natural products have demonstrated multi-pathway and multi-target therapeutic potential, with notable efficacy in precision antifibrotic intervention (71).

The limitations of this study are as follows: (1) the use of animal models and the absence of evidence at the protein phosphorylation level. Future studies will include optimized experimental designs to obtain protein-level evidence, as well as studies in human cell models and preliminary clinical trials to elucidate the mechanisms by which HSYA alleviates DN and to accelerate its clinical translation. (2) The results of molecular docking and simulations are primarily based on computational predictions and lack direct experimental evidence of physical binding. Future work will further evaluate the direct inhibition of the PI3K/AKT/mTOR pathway by HSYA and the associated physical binding to pathway components using *in vitro* kinase activity assays and physical binding analyses (surface plasmon resonance or isothermal titration calorimetry), thus assessing the gap between computational predictions and functional evaluation. (3) This study primarily focused on validating the core signaling pathways. Further investigation of the remaining predicted targets will be undertaken in future studies.

## 5 Conclusion

This study provides preliminary evidence for the therapeutic potential of HSYA in DN. This effect may involve the amelioration of fibrosis-related pathological changes in the kidney and the regulation of autophagy-related genes (PI3K, AKT, mTOR), thereby providing a theoretical basis for its development as a potential therapeutic agent.

## Data Availability

The datasets presented in this study can be found in online repositories. The names of the repository/repositories and accession number(s) can be found at: https://www.jianguoyun.com/p/Dc2WaX8Q5aDtDRip0I8GIAA.

## Footnotes

1
https://pubchem.ncbi.nlm.nih.gov/

2
https://www.swisstargetprediction.ch/

3
https://www.lilab-ecust.cn/pharmmapper/

4
https://www.uniprot.org/

5
https://www.genecards.org/

6
https://www.omim.org/

7
https://metascape.org/

8
https://string-db.org/

9
https://pubchem.ncbi.nlm.nih.gov/
